# Improving referrals from primary care to secondary mental health services through an educational intervention: experience from Qatar

**DOI:** 10.1192/bji.2021.5

**Published:** 2021-08

**Authors:** Ovais Wadoo, Sami Ouanes, Mohamed Ali Siddig Ahmed, Iman Saeed Ahmed Saeid, Samya Ahmad AlAbdulla, Majid AlAbdulla

**Affiliations:** 1 Senior Consultant, Department of Psychiatry, Hamad Medical Corporation, Qatar. Email: souanes@hamad.qa; 2 Clinical Fellow, Department of Psychiatry, Hamad Medical Corporation, Qatar; 3 Senior Consultant, Department of Psychiatry, Hamad Medical Corporation, Qatar; 4 Department of Psychiatry, Hamad Medical Corporation, Qatar; 5 Senior Consultant and Executive Director of Operations, Family Medicine, Primary Health Care Corporation, Qatar; 6 Senior Consultant and Chairman, Department of Psychiatry, Hamad Medical Corporation, Qatar

**Keywords:** Primary care, referral, pathway, collaboration, community mental health teams

## Abstract

Primary care is geared to manage patients with mild to moderate presentations of common mental disorders and to refer patients with more severe mental disorders to specialist mental health services. With growing demand for specialty care, the quality of the referral is increasingly important to ensure efficient patient flow across the primary/secondary care interface and appropriate use of secondary services. We report on an initiative in a Qatari mental health clinic to improve the quality of referrals from primary care to specialist mental health services through an educational intervention for family physicians. We highlight the problem, the intervention and the outcome of our initiative, which was the first of its kind in the region. The number of inappropriate referrals fell by 93%, and the number of referrals with inadequate clinical information declined from 15 (January 2019) to 1 (September 2019). Feedback was very positive; respondents reported feeling supported, with better understanding of care pathways, the scope of primary care and mental health services.

Qatar is a peninsula situated halfway down the western coast of the Arabian Gulf, bordered to the south by the Kingdom of Saudi Arabia. Qatar has a population of 2.7 million^1^ and is one of the world's wealthiest nations in terms of per capita gross domestic product.

Since the launch of the National Health Strategy and the National Mental Health Strategy mental health has been identified as a priority area.^2^

Primary and secondary care in Qatar is state funded. Primary care is provided by Primary Health Care Corporation (PHCC) and secondary care by Hamad Medical Corporation (HMC). Primary care physicians are generally seen as the gate-keepers to secondary care services.^3^ In Qatar, it is no different and PHCC provides primary healthcare services through an organised network of country-wide health centres.^4^ Our team receives electronic referrals from primary care. Referrals are triaged weekly by local consultants on the basis of the information provided by the referring primary care physician. Based on the referrals we receive at West Doha mental health clinic, we identified that there was scope for improvement of referrals to address the significant discrepancies in referral thresholds between primary care physicians.

## The problem

The interface between primary and specialty care centres on the referral. With growing demand for specialty care, referral quality is increasingly important to ensure efficient patient flow across the primary/secondary care interface; referrals should be necessary, appropriate, timely and well communicated.^5^ Each health organisation has its own factors that require analysis within the organisational context in order to improve the quality of referrals. We looked systematically at the quality of referrals to our specialist community clinic. We evaluated the necessity of the referral, the appropriateness of the destination and the quality of the referral. Our findings revealed that there were many referrals that had insufficient information from the referring doctor.^4,6^ There was little justification for urgent referrals. There were referrals that did not conform to the clinical guidelines and were well within the scope of the primary care physician. Some referrals were wrongly sent to our service, instead of being directed to services other than West Doha mental health clinic. This is no different from what has been reported globally. There is evidence from international literature reporting significant discrepancies in referral thresholds between primary care physicians.^7,8^ It is established that severity of illness is not the only factor considered for making a referral to specialist services. The discrepancies have been attributed to the experience of the primary care physician and the culture of the primary care practice. Other factors, such as the patient's gender and the doctor–patient relationship, have also been reported to influence referrals.^7,8^

### Primary/secondary care interface for mental health in Qatar

In Qatar, each health centre is allocated a lead physician as a nominated mental health champion. The mental health champion is a family physician with special interest in mental health, who went through an additional 6-month training in mental health and who can help other family physicians manage cases related to mental health. Primary care is geared to managing patients with mild to moderate presentations of common mental disorders and to refer patients with more severe mental disorders to secondary mental health services.^9^ To make it easier to clarify patient need and manage the primary/secondary care interface, there are referral guidelines in place recommending a stepped care approach to the management of mental disorders.^10,11^ HMC is the main public provider of secondary care mental health services. West Doha Mental Health Clinic is a part of HMC community mental health services. Our team offers psychiatric out-patient clinics for a defined catchment served by ten health centres.

## The intervention

There are many well-known referral management strategies with interventions that target primary care, specialist services or infrastructure. Some of the interventions known to improve the quality of referrals are based on effective collaboration between primary and secondary care, which ensures that the right patients receive the right care, in the right setting and at the right time.^12,13^

We initiated a project to enhance collaboration by conducting mental health educational meetings with family physicians. The guidelines used as a reference were the local Qatari guidelines for the management of anxiety and depression.^10,11^ We conducted meetings at the ten health centres and trained 109 PHCC physicians. This initiative is the first of its kind within the mental health services in this region.^6^ These meetings were led by psychiatrists and supported by mental health champions linked to each practice. During these meetings, we used structured feedback questionnaires that we created to collect data about physician demographics, qualifications, years of experience, the training the physicians have received for management of mental disorders and their previous experience of managing mental disorders. It became evident that primary care physicians in Qatar come from diverse backgrounds, with varying levels of training and experience. During these meetings we got an insight into primary care physicians' experiences with the referral process and the barriers they face in making high-quality referrals.

The main problem identified by a significant number of primary care professionals was lack of clarity regarding the remit of primary care and the referral process. Some of the primary care physicians identified a need for ongoing clinical training to help them gain confidence in managing common mental disorders. We conducted training sessions which were interactive and were tailored to the needs of the group. The topics included referral guidelines, the scope of primary and secondary care services, challenges in primary care, resource availability in primary care (such as psychology support clinics, psychotropic medication), care pathways, screening tools used, emergency psychiatric services and risk assessment, and stepped care intervention.^14,15^ The target audience was primary care physicians. The meetings were held on weekdays during the physicians’ allocated continuing professional development (CPD) time to maximise participation at the respective health centres. The frequency and duration of the meetings were decided in accordance with availability in each centre. We also initiated a process of providing regular feedback to primary care physicians following the triage of referrals. The feedback was through email and included clinical management advice as considered appropriate. It is established that receiving feedback from the specialist providers empowers the professional relationship between primary care physicians and specialists. It improves the coordination between different levels of medical care and ensures continuity of care.^7^

## Outcomes

**Fig. 1:**
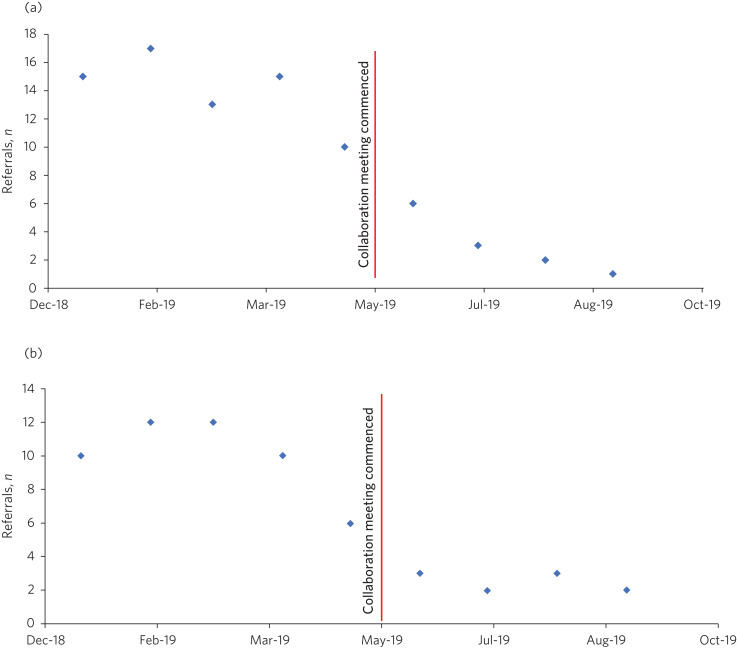
Referrals to secondary mental health services before and after the intervention. (a) Number of referrals with inadequate clinical information. (b) Number of referrals that should have been directed to other services (not to West Doha mental health clinic).

A qualitative review of referrals following the educational intervention indicated that there was improvement in volume and quality of referrals. There was a 93% reduction in the number of inappropriate referrals, with the number of referrals with inadequate clinical information declining from 15 (January 2019) to 1 (in September 2019, following the intervention). Similarly, the number of referrals that should have been directed to other services (other than West Doha mental health clinic) decreased by 80%, from 10 (January 2019) to 2 (in September 2019, following the intervention) (Fig. 1). This allowed for appropriate utilisation of secondary services, which in turn created capacity within our team to manage more complex cases in a timely manner. We collected feedback from the participating primary care physicians. The feedback was very positive (Table 1), and respondents reporting feeling supported, with better understanding of care pathways, the scope of primary care and mental health services. This educational intervention offered a platform for the primary care professionals to build communication channels, which enabled interaction between front-line family physicians and psychiatrists. The meetings created opportunities for less connected family physicians who had lacked interest or conviction about empowering mental health at the primary care level. The meetings allowed for mutual learning and facilitating improvement in the patient journey from primary to secondary care. These meetings ensured ownership, responsibility and linking the interventions to the severity of the problem and the skills of the provider. They also helped increase the skills and competence of primary care to deliver effective mental healthcare.

**Table 1:** Primary care physicians involved in the intervention: characteristics and feedback

Country of basic qualification, *n* (%)	
Arab country	50 (47.6)
Europe	17 (16.2)
Indian subcontinent	33 (31.4)
Other	5 (4.8)
Country of specialty qualification, *n* (%)	
Arab country	24 (22.0)
Europe	51 (46.8)
Indian subcontinent	29 (26.6)
Other	5 (4.6)
Total experience, years: mean (s.d.)	16.0 (9.0)
Total experience in Qatar, years: mean (s.d.)	7.7 (8.7)
Previous experience in mental health, *n* (%)	
No	30 (28.8)
Yes	74 (71.2)
‘Do you believe that primary healthcare has a role in mental health?’, *n* (%)	
No	6 (5.5)
Yes	103 (94.5)
‘Are you aware of the care pathways?’, *n* (%)	
No	20 (20.0)
Partly	18 (18.0)
Yes	62 (62.0)
‘Do you think you need more training in mental health?’, *n* (%)	
No	22 (21.6)
Yes	80 (78.4)
‘Which topics do you need additional training for?’, *n* (%)	
Anxiety	20 (31.7)
Depression	19 (30.2)
Psychotropic medication	12 (19.0)
Referral pathways	12 (19.0)
‘Did you find this session useful?’, *n* (%)	
No	1 (1.1)
Partly	3 (3.2)
Yes	89 (95.7)

## Challenges

It is difficult for primary care management to release family physicians for such sessions, as it can lead to service disruption. This was overcome by providing sessions in health centres and incorporating these sessions into the physicians’ allocated CPD time. The primary care physicians were initially sceptical about such projects, thinking them futile. This was addressed by providing interactive sessions based on participants’ needs and ensuring ongoing support by our services.

## Implications

The outcome of our study shows that a simple intervention can significantly improve the quality of referrals from primary to secondary mental health services. This can help enhance the quality of care provided to patients with mental health problems, and at the same time ensure an optimal use of the available resources.^15^
